# Enhanced photoelectrochemical and photocatalytic behaviors of MFe_2_O_4_ (M = Ni, Co, Zn and Sr) modified TiO_2_ nanorod arrays

**DOI:** 10.1038/srep30543

**Published:** 2016-07-28

**Authors:** Xin Gao, Xiangxuan Liu, Zuoming Zhu, Xuanjun Wang, Zheng Xie

**Affiliations:** 1 High-Tech Institute of Xi’an, Xi’an, 710025, China

## Abstract

Modified TiO_2_ nanomaterials are considered to be promising in energy conversion and ferrites modification may be one of the most efficient modifications. In this research, various ferrites, incorporated with various cations (MFe_2_O_4_, M = Ni, Co, Zn, and Sr), are utilized to modify the well aligned TiO_2_ nanorod arrays (NRAs), which is synthesized by hydrothermal method. It is found that all MFe_2_O_4_/TiO_2_ NRAs show obvious red shift into the visible light region compared with the TiO_2_ NRAs. In particular, NiFe_2_O_4_ modification is demonstrated to be the best way to enhance the photoelectrochemical and photocatalytic activity of TiO_2_ NRAs. Furthermore, the separation and transfer of charge carriers after MFe_2_O_4_ modification are clarified by electrochemical impedance spectroscopy measurements. Finally, the underlying mechanism accounting for the enhanced photocatalytic activity of MFe_2_O_4_/TiO_2_ NRAs is proposed. Through comparison among different transition metals modified TiO_2_ with the same synthesis process and under the same evaluating condition, this work may provide new insight in designing modified TiO_2_ nanomaterials as visible light active photocatalysts.

In recent years, titanium oxide (TiO_2_), as a “green” photocatalyst, has attracted lots of attention worldwide due to its low cost, nontoxicity and excellent photochemical stability^1^. However, due to the rapid recombination of photogenerated electron-hole pairs as well as the lack of visible light absorption^2^, the photocatalytic and photoelectrochemical efficiency of pure TiO_2_ is quite limited. Pure TiO_2_, with bandgap of *ca*. 3.0 to 3.2 e*V*, can only be excited under UV light irradiation, which comprises only ~5% of the total solar radiation, so, application of the unmodified TiO_2_ in solar energy conversion is far limited^3^. Besides, the excited charge carriers in TiO_2_ can recombine quickly and more than 90% of the recombination processes take place in 10 *ns*^4^, therefore, only a little fraction of the excited carriers can transfer to the surface of TiO_2_ and take part in the following photocatalytic process. To make full use of solar energy and reduce the recombination rate, many attempts have been made, such as doping with metal/nonmetal atoms and deposition of metals. Though the aforementioned methods can partly improve the photocatalytic as well as photoelectrochemical activity of TiO_2_, some problems remain unresolved. For example, doped TiO_2_ may suffer from thermal instability, photo corrosion, lattice distortion, while metal loading may result in an increase in the carrier-recombination probability^5^. In this case, one of the promising strategies is to couple TiO_2_ with other narrow band gap semiconductors, which are capable of harvesting photons in the visible light region. Recently, CdS with a low band gap (*ca.* 2.4 eV) was studied intensively to improve the visible light uitilizition of TiO_2_. However, its application is also hindered due to the low photostability^6^. In view of this, transition metal ferrites with molecular formula of MFe_2_O_4_ (M = Zn, Co, Ni, etc.) go into the vision of researchers considering its outstanding attributes. Firstly, these materials possess an important characteristic of narrow band gap, which could absorb the visible light efficiently^7^ and thus promote the photocatalytic reactions. Secondly, MFe_2_O_4_ has a good electrical conductivity due to the electron hopping process between different valence states of metals in O-sites, which is beneficial for the transfer of charge carriers^8^,^9^. Furthermore, transition metal ferrites have many intriguing advantages such as well stability against photocorrosion, good superparamagnetic properties, low toxicity, easy preparation, high adsorption ability, low cost, and abundant resources^10^,^11^,^12^. So far, most of the researches are focused on separation of TiO_2_ powders from treated water by employing its magnetic property of transition metal ferrites^13^,^14^,^15^. It is scarce on study of the visible responsiveness of MFe_2_O_4_ to increase utilization of solar energy as well as to enhance the photoelectrochemical and photocatalytic performance of TiO_2_.

ZnFe_2_O_4_, with a relatively small band-gap (*ca.* 1.9 eV)^16^, is the most frequently studied to modify TiO_2_ in enhancing the photoelectrochemical capacity. Yuan et.al observed that the ZnFe_2_O_4_/TiO_2_ nanocomposite is more effective as a photocatalyst in the phenol degradation than pure TiO_2_. However the mechanism of the enhanced photoactivity of the ZnFe_2_O_4_/TiO_2_ composite is still needed to be further understood^17^. Furthermore, the following researches proposed similar theory to explain the role of ZnFe_2_O_4_ in enhancing photoactivity of TiO_2_^18^,^19^, that is, the adoption of ZnFe_2_O_4_ makes the ZnFe_2_O_4_/TiO_2_ composite could use visible light, and the good match of band edges between ZnFe_2_O_4_ and TiO_2_ is in favor of charge carriers separating effectively. Reports about other MFe_2_O_4_ modified TiO_2_, such as NiFe_2_O_4_/TiO_2_, Mn_0.5_Zn_0.5_Fe_2_O_4_/TiO_2_, MgFe_2_O_4_/TiO_2_ and CuFe_2_O_4_/TiO_2_ all show higher photoelectrochemical and photocatalytic performance^20^,^21^,^22^,^23^. It seems that MFe_2_O_4_ is such a promising material to improve the photoelectrochemical and photocatalytic performance of TiO_2_. However, the comparison among the photoelectrochemical and photocatalytic performances of transition metal ferrites modified TiO_2_ reported in the literatures is extremely difficult, because the experimental conditions were very different, such as catalysts synthesis process, light irradiation wavelength, reactor geometric configuration, catalyst loading and so on. Moreover, the origin and the crystalline structure of TiO_2_, which strongly affect its electronic and photoactivity, are also different. Therefore, the same condition should be taken into consideration when assessing the real effect of transition metal ferrites on the photoactivity of TiO_2_, such as using the same bare TiO_2_ as the starting material, taking the same procedure to modify TiO_2_ by transition metal ferrites, and finally evaluating their performances with unified standards. NiFe_2_O_4_, CoFe_2_O_4_, ZnFe_2_O_4_ and SrFe_2_O_4_ are four common transition metal ferrites which have been frequently studied with their magnetism, but except for ZnFe_2_O_4_, the other three are not common in modifying TiO_2_ to enhance its photoactivity, therefore, we select the four as research objects, making a comparision between the common one (ZnFe_2_O_4_/TiO_2_) and the uncommon ones (CoFe_2_O_4_, ZnFe_2_O_4_ and SrFe_2_O_4_ modified TiO_2_).

In addition to incorporate other materials to modify TiO_2_, structural design is another important method to enhance the photoactivity of TiO_2_. One-dimensional (1D) nanostructure such as nanowire, nanotube, nanorod have attracted lots of attention due to the unique physical and chemical properties. 1D TiO_2_ nanomaterials possess all the typical features of TiO_2_ nanoparticles^24^. Electron diffusion length (up to ~100 *μm*) can be prolonged by using vertically aligned 1D nanostructures and excited electrons can easily pass along 1D nanostructure to the transparent conducting oxide electrode^25^,^26^, which facilitate charge transfer and promote charge separating efficiently^20^,^27^. However, the relatively low specific surface area on a smooth surface of 1D nanostructures may decrease the absorption ability and a single crystal phase of 1D nanostructures may pose certain constraints on the photoelectrochemical performance^24^,^28^. Fortunately, these disadvantages can be surmouned by introducing the second phase, i.e., doping metals/nonmetals or forming heterjunctions. Among1D nanostructures, 1D nanorod arrays with large area can be easily obtained by hydrothermal method, which is facile, economic and controllable^29^. Therefore, coupling the traits of one-dimensional TiO_2_ nanorods (TiO_2_ NRAs) and visible light responsive MFe_2_O_4_ nanoparticles seems to be a promising way to enhance the solar energy conversion efficiency of TiO_2_.

To the best of our knowledge, there is few systematic research on the photoelectrochemical and photocatalytic capacity of various MFe_2_O_4_ modified one-dimensional TiO_2_ NRAs so far. In this study, large area uniform TiO_2_ NRAs were synthesized hydrothermally and ferrites containing vaious cations (MFe_2_O_4_, M = Ni, Co, Zn, and Sr) were utilized to modify the as-prepared TiO_2_ NRAs. The morphology, crystalline structures and optical properties as well as photoelectrochemical performances of TiO_2_ NRAs and MFe_2_O_4_/TiO_2_ NRAs were investigated. Moreover, the photocatalytic activities of the MFe_2_O_4_/TiO_2_ NRAs were evaluated in the degradation of Cr(VI) aqueous solution under visible light irradiation. Finally, the underlying photcatalytic mechanism was discussed.

## Results and Discussion

**Figure 1:**
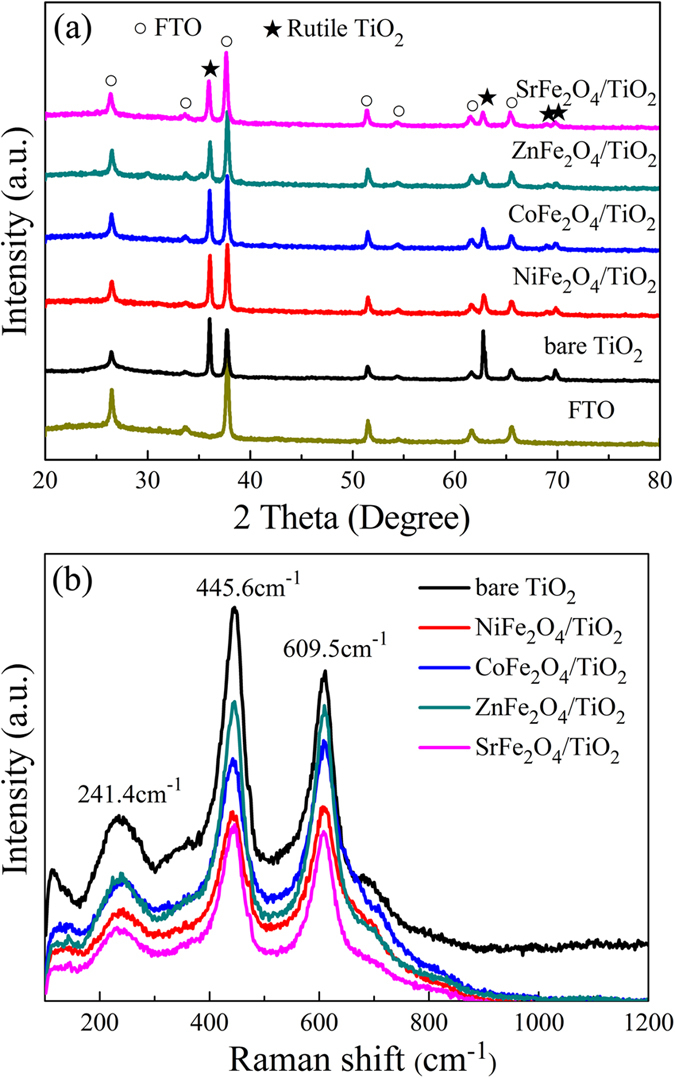
(**a**) XRD patterns and (**b**) Raman spectra for TiO_2_ NRAs and MFe_2_O_4_/TiO_2_ NRAs.

Figure 1(a) displays the XRD patterns collected from the TiO_2_ NRAs and MFe_2_O_4_/TiO_2_ NRAs. It can be seen that the TiO_2_ NRAs and MFe_2_O_4_/TiO_2_ NRAs all features the characteristic peaks at 2*θ* = 36.078°, 62.750°, 69.010° and 69.795°, indicative of rutile TiO_2_ (PDF NO. 21–1276). Other peaks can be attributed to the diffraction of FTO substrate. There is no typical diffraction peaks of MFe_2_O_4_ after modification, which may be owing to the low content of MFe_2_O_4_. The content of MFe_2_O_4_ will be discussed in the following SEM characterization.

In order to further examine the phase composition of the samples and confirm the existence of MFe_2_O_4_, Raman spectroscopy was employed. As is shown clearly in Fig. 1(b), there are three Raman peaks at 241.4, 445.6 and 609.5 cm^−1^ for all samples, which are assigned to the Raman active modes of rutile^30^. This result indicates that the rutile phase dominates the crystalline structure of the samples, which is in accordance with the XRD result. However, the Raman peak corresponding to MFe_2_O_4_ is not discernable due to the low content of MFe_2_O_4_. The peak at 117 cm^−1^ is due to plasma emission of the Ar^+^ laser^31^.

**Figure 2:**
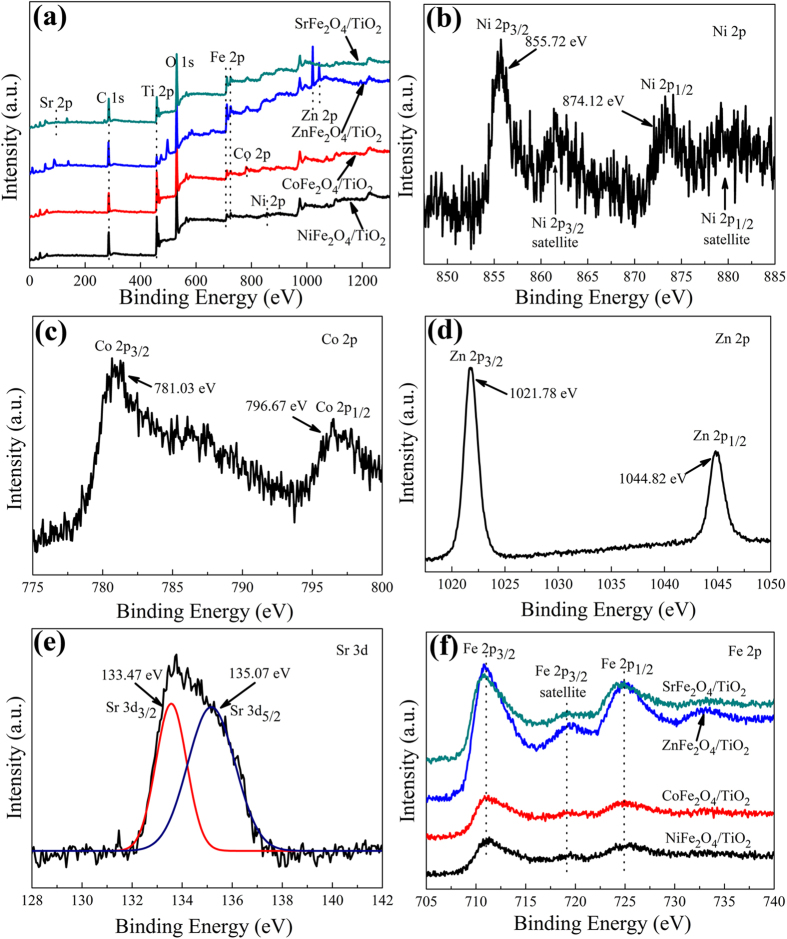
(**a**) XPS survey spectra of MFe_2_O_4_/TiO_2_ NRAs and high-resolution XPS spectra of (**b**) Ni 2p, (**c**) Co 2p, (**d**) Zn 2p, (**e**) Sr 2p and (**f**) Fe 2p.

In order to further confirm the existence of MFe_2_O_4_, XPS measurement was carried out. The XPS survey spectra are shown in Fig. 2(a). The peaks located at the binding energies of *ca.* 458–464 eV, 529–531 eV, 711–725 eV and 284–288 eV in all samples are ascribed to the Ti 2*p*, O 1*s*, Fe 2*p*, and C 1*s*, respectively. On the other hand, these MFe_2_O_4_/TiO_2_ NRAs materials also show their characteristic peaks located between 850 and 875 eV (Ni 2*p*) for NiFe_2_O_4_/TiO_2_ NRAs, 781 and 796 eV (Co 2*p*) for CoFe_2_O_4_/TiO_2_ NRAs, 1021 and 1044 eV (Zn 2*p*) for ZnFe_2_O_4_/TiO_2_ NRAs, and 134 eV (Sr 2*p*) for SrFe_2_O_4_/TiO_2_ NRAs.

As is shown in Fig. 2(b), the Ni 2*p* peaks of NiFe_2_O_4_/TiO_2_ NRAs consist of two characteristics of Ni 2*p*3/2 (855.72 eV) and Ni 2*p*1/2 (874.12 eV)^32^, indicative of the presence of Ni^2+^. Similar to the Ni 2*p* peaks in NiFe_2_O_4_/TiO_2_ NRAs, the Co 2*p* XPS spectra recorded from the CoFe_2_O_4_/TiO_2_ NRAs sample, containing Co 2*p*3/2 (781.03 eV, Co^2+^ in Tet-site) and Co 2*p*1/2 (796.67 eV, Co^2+^ in Tet-site), indicate that Co^2+^ exists in the CoFe_2_O_4_/TiO_2_ NRAs according to the literature reports^33^. For the ZnFe_2_O_4_/TiO_2_ NRAs, the recorded Zn 2*p* XPS spectra indicate that Zn^2+^ exists in the ZnFe_2_O_4_/TiO_2_ NRAs, which is also consistent with literature reports^34^,^35^. Furthermore, the manganese valences were determined by the position of the multiplet splitting of Sr 2*p* peaks, the positions of Sr 2*p*3/2 and Sr 2*p*1/2 were all assigned to Sr^2+^. As for high-resolution XPS spectra of Fe 2*p* in Fig. 2(d), one can see that the peaks at *ca*. 711.6 eV and *ca*. 724.9 eV can be attributed to Fe 2*p*3/2 and Fe 2*p*1/2 for Fe^3+^, respectively, which reveals the oxidation state of Fe^3+^ in the MFe_2_O_4_/TiO_2_ heterostructure^33^,^36^.

**Figure 3:**
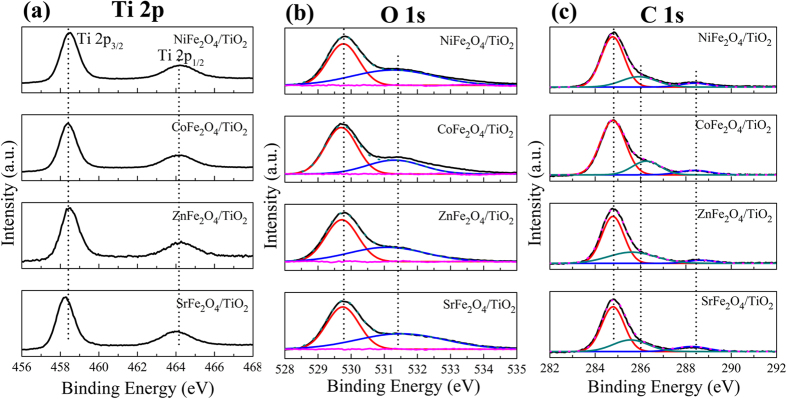
High-resolution XPS spectra of (**a**) Ti 2p, (**b**) O 1s and (**c**) C 1s.

The high resolution XPS spectra of Ti 2*p*, O 1*s*, and C 1*s* are shown in Fig. 3. The Ti 2*p* spectra, as presented in Fig. 3(a), all show the main peak located at *ca*. 458.5 eV and *ca*. 464.2 eV, which can be attributed to Ti 2*p*3/2 and Ti 2*p*1/2 in TiO_2_, respectively^37^. It is clear that the O 1*s* spectra of these MFe_2_O_4_/TiO_2_ NRAs samples can be deconvoluted into two components centered at *ca*. 529.8 eV and *ca*. 531.4 eV using two Gaussian curve fittings {Fig. 3(b)}, The components at the lower and higher binding energy side can be assigned to the crystal lattice oxygen of TiO_2_ and MFe_2_O_4_ and chemisorbed oxygen in a defective lattice site (i.e.–OH), respectively^32^,^38^,^39^,^40^,^41^. It is suggested that the hydroxyl group can capture the photogenerated holes and form highly reactive hydroxyl free radicals, which plays an important role in enhancing photocatalytic activity^18^. The high resolution XPS spectrum of C 1*s* is shown in Fig. 3(c). The primary peak located at *ca*. 284.6 eV is assigned to C–C/C–H bonds from adventitious carbon^42^, while the peaks at *ca*. 286.2 eV and *ca*. 288.4 eV can be attributed to the formation of carbonate species, resulting mainly from CO_2_ adsorption^38^,^43^,^44^,^45^. Especially, the peak at 288.4 eV can be ascribed to the Ti–O–C structure in carbon doped TiO_2_ by substituting some of the lattice titanium atoms^46^,^47^,^48^. Interestingly, carbon doping is beneficial to light absorption capability as well as absorption of organic molecules to some extent^24^,^25^.

**Figure 4:**
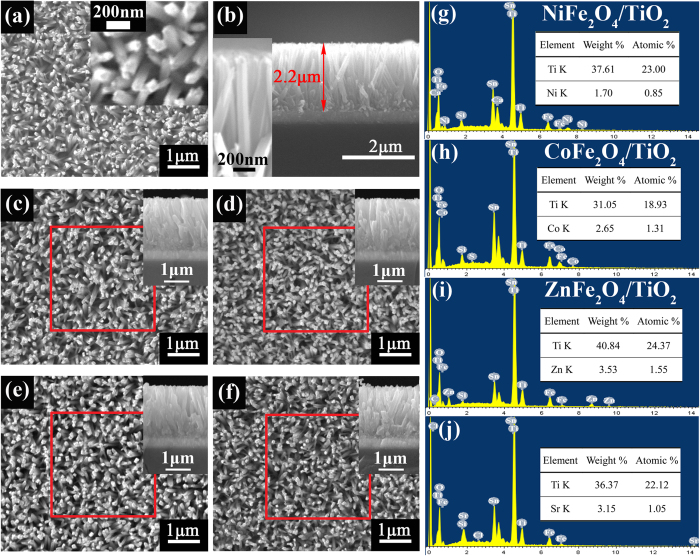
SEM images of (**a**) the bare TiO_2_ NRAs, (**b**) the cross section image of the bare TiO_2_ NRAs, (**c**) NiFe_2_O_4_/TiO_2_ NRAs, (**d**) CoFe_2_O_4_/TiO_2_ NRAs, (**e**) ZnFe_2_O_4_/TiO_2_ NRAs and (**f**) SrFe_2_O_4_/TiO_2_ NRAs. The insets of (**c**–**f**) are the corresponding cross section images. (**h**,**i**,**g**,**k**) are the EDS results of the red square region in (**c**–**f**), respectively.

The SEM images of the bare TiO_2_ NRAs and MFe_2_O_4_/TiO_2_ NRAs are shown in Fig. 4. It is noteworthy that, after MFe_2_O_4_ modification as shown in Fig. 4(c–f) from the top view images, the samples have no obvious changes in morphology compared with the bare TiO_2_ NRAs in Fig. 4(a), which indicates that the deposited MFe_2_O_4_ nanoparticles are of extremely fine size. The vertically or slantingly aligned TiO_2_ nanorods arrays, with diameter of 60~120 nm and length of 2.2 *μ*m, are grown homogeneously on FTO substrate with rectangular cross section. In order to measure the content of MFe_2_O_4_ in MFe_2_O_4_/TiO_2_ NRAs heterjunction, energy dispersive x-ray spectrum (EDS) analysis was carried out. The results, shown in Fig. 4(g~j), are obtained from collecting the EDS data in red square region of the MFe_2_O_4_/TiO_2_ NRAs in Fig. 4(c–f), respectively. It is confirmed that Ni, Co, Zn and Sr are present in NiFe_2_O_4_, CoFe_2_O_4_, ZnFe_2_O_4_ and SrFe_2_O_4_ modified TiO_2_ NRAs, respectively. Indeed, only a trace amount of Ni, Co, Zn and Sr can be observed in these samples.

**Figure 5:**
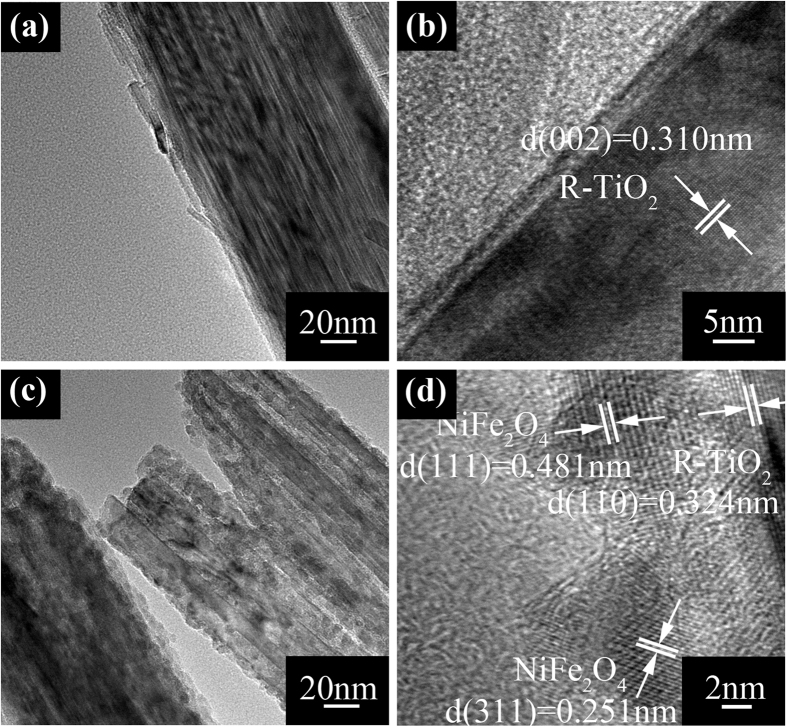
(**a**,**b**) TEM images of TiO_2_ NRAs, (**c**,**d**) TEM images of NiFe_2_O_4_/TiO_2_ NRAs.

Furthermore, structural characterizations of the MFe_2_O_4_ modified TiO_2_ nanorods were investigated by TEM. Figure 5(a) shows the TEM image of the bare TiO_2_ nanorod. Essentially, the diameter of the bare TiO_2_ nanorod under TEM observation is consistent with the SEM result. It can be seen clearly that the bare TiO_2_ nanorod is very smooth. After MFe_2_O_4_ modification shown in Fig. 5(c), the nanorod surface becomes rough, and the ultrafine NiFe_2_O_4_ particles, with diameter of *ca*. 3~5** **nm as shown in Fig. 5(d), are uniformly deposited on the nanorod. In addition, the high resolution HRTEM image gives lattice fringes of about 0.481** **nm and 0.251** **nm, corresponding to the d (111) and d (311) space of NiFe_2_O_4_, respectively. Analysis of TEM was also applied to CoFe_2_O_4_, ZnFe_2_O_4_ and SrFe_2_O_4_ modified TiO_2_ NRAs (shown in Supplementary Fig. S1), and all show the same morphology. i.e., the smooth surface of TiO_2_ nanorod become rough after MFe_2_O_4_ modification. The corresponding lattice fringes of CoFe_2_O_4_, ZnFe_2_O_4_ and SrFe_2_O_4_ are shown in Figure S1(b,d,f), respectively.

**Figure 6:**
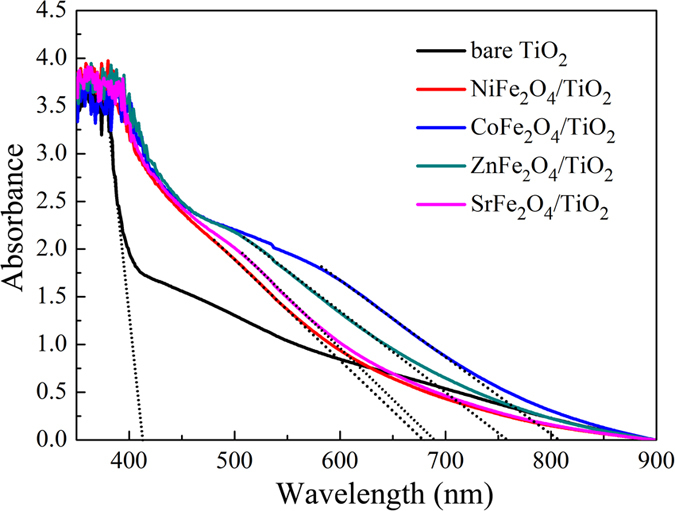
Absorption spectra of MFe_2_O_4_/TiO_2_ NRAs. The dash lines are the extension of the linear portion of absorbance.

The optical absorption spectra of TiO_2_ NRAs and MFe_2_O_4_/TiO_2_ NRAs are shown in Fig. 6. All samples exhibit typical UV absorption (λ < 380 nm). It is noteworthy that, compared with bare TiO_2_ NRAs, all MFe_2_O_4_/TiO_2_ samples exhibit strong light absorption in a wide region from 380 nm to 900 nm, which can be attributed to the intrinsic band gap absorption of MFe_2_O_4_. However, unlike other pure TiO_2_, tiny absorption of the as-prepared TiO_2_ sample in the visible light range can be observed. There are two reasons accounting for this abnormal phenomenon, one is the scattering of light caused by the nanorod arrays, and the other is the impurity doping during the hydrothermal and sintering process^49^,^50^,^51^. The absorption capacity of CoFe_2_O_4_/TiO_2_ NRAs is the biggest, followed by ZnFe_2_O_4_, SrFe_2_O_4_ and NiFe_2_O_4_ modified TiO_2_ NRAs sequentially. The corresponding band gaps are calculated from the plots of *E*_g_ = 1240/*λ* by extrapolating the linear portion of absorbance to the wavelength axis where absorbance is zero^52^. As is shown in Fig. 6, the steep absorption edge of the bare TiO_2_ NRAs locates at about 410 nm, corresponding to band gap (*E*_g_) of about 3.02 eV. MFe_2_O_4_ modified TiO_2_ NRAs samples all exhibit red-shift with smaller band gaps compared with bare TiO_2_ NRAs, and the *E*_g_ is *ca*. 1.84 eV, 1.63 eV, 1.81 eV and 1.53 eV for NiFe_2_O_4_/TiO_2_ NRAs, ZnFe_2_O_4_/TiO_2_ NRAs, SrFe_2_O_4_/TiO_2_ NRAs, and CoFe_2_O_4_/TiO_2_ NRAs, respectively.

**Figure 7:**
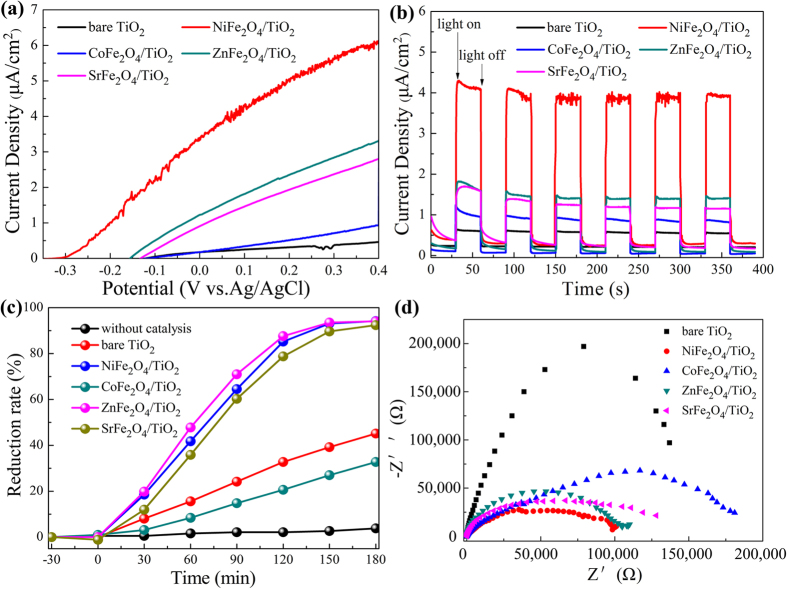
(**a**) Photocurrent density versus potential of the MFe_2_O_4_/TiO_2_ NRAs, (**b**) Photocurrent density versus time measurements of MFe_2_O_4_/TiO_2_ NRAs under 0 V versus Ag/AgCl bias, (**c**) Photocatalytic reduction of Cr(VI) by MFe_2_O_4_/TiO_2_ NRAs under visible light, (**d**) Nyquist plots of the EIS spectra of MFe_2_O_4_/TiO_2_ NRAs.

To evaluate the effect of MFe_2_O_4_ modification on the photoelectrochemical properties of TiO_2_ NRAs, the photocurrent intensity versus potential (*I*–*V*) and photocurrent density versus time (*I*-*T*) measurements of MFe_2_O_4_/TiO_2_ NRAs were performed. The *I*-*V* characteristics of MFe_2_O_4_/TiO_2_ NRAs are shown in Fig. 7(a). The photocurrent density in dark can be neglected for all samples. Under visible light irradiation, the photocurrent density of bare TiO_2_ NRAs varies little with increase in bias potential, while the photocurrent density of MFe_2_O_4_/TiO_2_ NRAs increases significantly at more positive bias potentials, except for CoFe_2_O_4_/TiO_2_ NRAs with only a slight increase. For example, at bias potential of 0.4 V vs. Ag/AgCl, the photocurrent density of NiFe_2_O_4_, ZnFe_2_O_4_ and SrFe_2_O_4_ modified TiO_2_ NRAs is 6.13, 3.31 and 2.81 μA/cm^2^, respectively, while the photocurrent density of CoFe_2_O_4_/TiO_2_ NRAs is only 0.95 μA/cm^2^, which is far lower than that of other MFe_2_O_4_ modified samples, and only a little higher than that of the bare TiO_2_ NRAs (0.46 μA/cm^2^ at 0.4 V vs. Ag/AgCl). It is reported that the more negative open circuit potential (Voc) means better charge carrier separation and electron accumulation in semiconductor-semiconductor heterojunctions^53^,^54^,^55^,^56^. After MFe_2_O_4_ modification, Voc for NiFe_2_O_4_/TiO_2_ NRAs, ZnFe_2_O_4_/TiO_2_ NRAs and SrFe_2_O_4_/TiO_2_ NRAs is −0.323, −0.156 and −0.133 V, respectively, which becomes more negative than that of the bare TiO_2_ NRAs (−0.121 V), except for CoFe_2_O_4_ modified one(−0.117 V). From the varying trend of Voc, one can see that MFe_2_O_4_/TiO_2_ NRAs (M = Ni, Zn and Sr) heterjunction facilitates the separation and transfer of the charge carriers, while CoFe_2_O_4_/TiO_2_ NRAs is not favourable for charge carriers separation. Figure 7(b) plots the *I*-*T* characteristics of the MFe_2_O_4_/TiO_2_ NRAs. It is observed that all the samples exhibit a quick response to the on/off of the incident light, and the current density of MFe_2_O_4_ modified TiO_2_ NRAs shows an enhancement compared with that of bare TiO_2_ NRAs. NiFe_2_O_4_/TiO_2_ NRAs displays the biggest photocurrent density of ca. 4.13 μA/cm^2^, followed by ZnFe_2_O_4_, SrFe_2_O_4_ and CoFe_2_O_4_ modified ones, with 1.73, 1.68 and 1.01 μA/cm^2^, respectively. The enhancement induced by CoFe_2_O_4_ modification is relatively low, only 0.4 μA/cm^2^ higher than that of bare TiO_2_ NRAs (0.61 μA/cm^2^). The changing trend of *I-T* result is consistent with the *I-V* characteristics of the MFe_2_O_4_/TiO_2_ NRAs.

Though all MFe_2_O_4_ modified TiO_2_ NRAs samples exhibit a broader and stronger absorption than the bare TiO_2_ NRAs (see Fig. 6), only NiFe_2_O_4_/TiO_2_ NRAs possesses a significant enhancement in PEC performance. Very limited improvement for CoFe_2_O_4_ modification may result from the inefficient separation of photoexcited charge carriers. This phenomenon is due to the fact that the conduction band (CB) of CoFe_2_O_4_ is more positive than that of TiO_2_, while the valence band (VB) of CoFe_2_O_4_ is more negative than that of TiO_2_^41^,^57^, which is not favour in carriers separating.

To investigate the photocatalytic capacity of the MFe_2_O_4_/TiO_2_ NRAs, experiments were carried out for Cr(VI) photoreduction under visible light irradiation. The concentration changes are detected by the absorption peak (365 nm) of Cr(VI) in the UV-vis spectrum. The photodegradation results are shown in Fig. 7(c). After irradiation for 180 minutes, little Cr(VI) was reduced without catalyst (the reduction rate is only 3.8%). Under the same condition, only 45.1% of Cr(VI) was reduced when bare TiO_2_ NRAs was used as a photocatalyst. However, the potoreduction capacity of NiFe_2_O_4_, ZnFe_2_O_4_ and SrFe_2_O_4_ modified TiO_2_ NRAs are enhanced greatly (94.18%, 94.086% and 92.39%, respectively), reaching the same level. This may be attributed to the function of citric acid serving as a sacrificial electron donator to quickly consume the photogenerated holes^19^, thus greatly promote charge separation and further improv photocatalytic reactions. Unfortunately, CoFe_2_O_4_ modification makes the photocatalytic degradation rate of Cr(VI) even lower. The following reason may account for this abnormal phenomenon. Eventhough CoFe_2_O_4_ modified TiO_2_ NRAs can be excited more easily under visible light irradiation, and then generates more charge carriers, the recombination rate of CoFe_2_O_4_/TiO_2_ NRAs seems to be higher than that of the bare TiO_2_ NRAs which can be deduced from the Voc changes, thus leading to the lower photocatalytic capacity of CoFe_2_O_4_/TiO_2_ NRAs.

In order to clarify the enhancement in the phototelectochemical and photocatalytic capacity of TiO_2_ NRAs after MFe_2_O_4_ modification, it is important to figure out the separating and transferring efficiency of the charge carriers, so electrochemical impedance spectroscopy (EIS) measurements were conducted. As shown in Fig. 7(d), except for CoFe_2_O_4_/TiO_2_ NRAs, other MFe_2_O_4_ modified TiO_2_ NRAs samples all have a smaller arc radius compared with that of the bare TiO_2_ NRAs. It is generally assumed that the smaller arc radius on the EIS Nyquist plot suggests a more effective separation of the photogenerated electron-hole pairs and a faster interfacial charge transfer^38^,^58^. From the EIS spectra, it can be seen clearly that NiFe_2_O_4_, ZnFe_2_O_4_ and SrFe_2_O_4_ modified TiO_2_ NRAs have a smaller arc radius than the bare TiO_2_ NRAs. It means that the charge carriers separate and transfer more effectively in NiFe_2_O_4_/TiO_2_ NRAs, ZnFe_2_O_4_/TiO_2_ NRAs and SrFe_2_O_4_/TiO_2_ NRAs, thus leading to the significant enhancement of the phototelectochemical and photocatalytic capacity of the modified TiO_2_ NRAs. While the arc radius of CoFe_2_O_4_/TiO_2_ NRAs is even bigger than that of the bare TiO_2_ NRAs, suggesting lower separating rate of charge carriers in CoFe_2_O_4_/TiO_2_ NRAs and thus resulting in the limited enhancement of the phototelectochemical capacity and even decrease in photocatalytic performance. This EIS result of CoFe_2_O_4_/TiO_2_ NRAs is in accordance with the Voc value of CoFe_2_O_4_/TiO_2_ NRAs in the *I-V* curves as well as the deduction from the band matching between CoFe_2_O_4_ and TiO_2_ in previous literature, that is, the CoFe_2_O_4_/TiO_2_ heterojunction is not conducive to effective separation of carriers.

**Figure 8:**
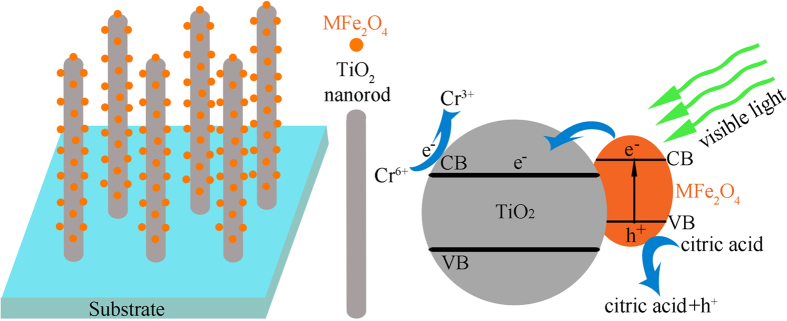
Photocatalytic schematic of Cr(VI) by MFe_2_O_4_/TiO_2_ NRAs.

Photocatalytic schematic of Cr(VI) by MFe_2_O_4_/TiO_2_ NRAs is shown in Fig. 8. Under visible light illumination, MFe_2_O_4_ is effectively excited to generate electrons and holes. Because the conduction band of MFe_2_O_4_ is more positive than that of TiO_2_, the excited electrons can quickly transfer from MFe_2_O_4_ to the conduction band of TiO_2_, whereas the generated holes accumulate in the valence band of MFe_2_O_4_. Consequently, the excited electron/hole pairs could be separated effectively, which contributes to the improvement of photoelectrochemical properties of MFe_2_O_4_/TiO_2_ NRAs, except for CoFe_2_O_4_/TiO_2_ NRAs. Due to the efficient separation of the photogenerated electrons and holes by MFe_2_O_4_ modification, the lifetime of the charge carriers are prolonged, leading to an efficient oxidation-reduction reaction, so the photodegradation activity can be enhanced. When the photoreduction is carried out in the presence of citric acid, it can quickly consume the accumulated holes in the valence band, and thus the electrons in the conduction band have enough time to function with the Cr(VI) in the aqueous solution.

## Conclusions

The effect of different ferrits (MFe_2_O_4_, M = Ni, Co, Zn and Sr) modification on improving the photoelectrochemical and photocatalytic properties of TiO_2_ have been probed. By changing the incorporated cations in the MFe_2_O_4_, we have found that NiFe_2_O_4_ modification can greatly enhance the photoelectrochemical and photocatalytic performance of TiO_2_ NRAs, while CoFe_2_O_4_ has relative limited effect. Compared with the bare TiO_2_ NRAs, the photocurrent density of NiFe_2_O_4_/TiO_2_ NRAs is twelve-fold higher in the I-V curve at 0.4 V vs. Ag/AgCl. Under visible light irradiation, the Cr(VI) photoreduction rate of NiFe_2_O_4_/TiO_2_ NRAs achieves one-fold higher than that of the bare TiO_2_ NRAs. The EIS measurement provides a clearer understanding of the role that MFe_2_O_4_ have in photogenerated charge carriers effectively separating and transferring. Except for CoFe_2_O_4_/TiO_2_ NRAs, other MFe_2_O_4_ modified TiO_2_ NRAs have more effective separation and transfer of the charge carriers, thus leading to the difference in the photoelectrochemical and photocatalytic performance of MFe_2_O_4_ modified TiO_2_ NRAs. The obtained results point that the visible active MFe_2_O_4_ modification may be a promising way to improve TiO_2_ for applications in photocatalytic activity as well as in photoelectrochemical conversion with solar light.

## Methods

### Materials synthesis

All reagents used were analytical grade chemicals and used without further treatment.

### Synthesis of MFe_2_O_4_ modified TiO_2_ nanorod arrays

Aligned TiO_2_ NRAs were vertically grown on transparent fluorine-doped tin oxide (FTO) substrates by the hydrothermal method. Deionized water (DI, 10 mL) was mixed with hydrochloric acid (36.8 wt%, 10 mL) and stirred for 10 min before tetrabutyl titanate (98%, 0.4 mL) was added. When the solution was stirred until clear clarification, it was transferred to a Teflon-lined stainless steel autoclave. Clean FTO substrates were immersed with the conducting side face down. The autoclave was put in an oven at a temperature of 150 °C and was taken out from the oven after 5 h. After the autoclave was cooled to room temperature, the FTO substrate was rinsed with DI water and dried naturally at room temperature. The final area of the nanorod arrays was approximately 4.5 cm^2^.

For the preparation of ZnFe_2_O_4_/TiO_2_ NRAs, briefly, zinc nitrate and iron nitrate were dissolved in DI water at room temperature to form a mixture, the as-prepared TiO_2_ NRAs were soaked in the Fe(NO_3_)_3_ and Zn(NO_3_)_2_ mixed solution (with concentrations of 0.25 M and 0.125 M, respectively) for 1 h, followed by dipping in DI water for 5s. Afterwards the nanorod arrays were dried in air for 24 h and then annealed at 500 °C in air for 2 h with heating and cooling rates of 5 °C·min^−1^. The MFe_2_O_4_/TiO_2_ NRs (M = Ni, Co and Sr) were prepared using the same method by replacing the zinc nitrate with other nitrate.

### Characterization

The surface morphology was obtained with a scanning electron microscopy (SEM, VEDAIIXMUINCN) equipped with an energy dispersive X-ray spectroscopy (EDS) system. The film microstructure was further characterized by transmission electron microscopy (TEM). X-ray diffraction (XRD, PANalytical) with Cu-Ka (λ = 0.15401 nm) was operated at 40 kV and 40 mA in a 2θ range of 20–80° at a scanning speed of 5° min^−1^ to characterize the crystal structure. Raman spectra were recorded at room temperature using a inVia Reflex Raman spectrometer under Ar^+^ (532 nm) laser excitation. The optical properties were probed by a UV–vis spectrophotometer (UV1800, Shimadzu) with a FTO substrate as a blank. X-ray photoelectron spectroscopy (XPS) was obtained using a ESCALAB 250Xi (The binding energy of the XPS spectra was calibrated with the reference to the C 1s peak at 284.8 eV.)

### Photoelectrochemical and photocatalytic measurement

photoelectrochemical measurements were performed in a 250 mL quartz cell using a three-electrode configuration, including the prepared samples as working electrode, a Pt foil as counter electrode, a saturated Ag/AgCl as reference electrode, and 0.5 M Na_2_SO_4_ aqueous solution as an electrolyte. The working electrode was illuminated within an area of about 1.5 cm^2^ at zero bias voltage versus the Ag/AgCl electrode under solar-simulated (AM 1.5 G filtered, 100 mW·cm^−2^, CEL-HXF300) light sources with a UV cutoff filter (providing visible light with λ ≥ 420 nm). The electrochemical impedance spectroscopy (EIS) measurements were recorded by employing an AC voltage of 5 mV amplitude with the initial potencial at 0.4 v (vs. Ag/AgCl) over the frequency range from 100 kHz to 100 mHz without light illumination.

The Cr(VI) photoreduction was performed in a quartz cell. In the photoreduction experiments, 15 mL of aqueous solution containing 20 mg·L^−1^ of K_2_Cr_2_O_7_ and 85 mg·L^−1^ of citric acid was used. The citric acid served as a sacrificial electron donator. Prior to irradiation, the photocatalyst (area about 6 cm^2^) was immersed into the Cr(VI) solution in the dark for 30 minutes to establish an adsorption/desorption equilibrium. The relative concentration of Cr(VI) in the solution was derived by comparing its UV–vis absorption intensity with that of the initial Cr(VI) solution at 365 nm. The light source was a 300 W xenon lamp with visible light illumination of 26.5 mW·cm^−2^.

## Additional Information

**How to cite this article**: Gao, X. *et al*. Enhanced photoelectrochemical and photocatalytic behaviors of MFe_2_O_4_ (M = Ni, Co, Zn and Sr) modified TiO_2_ nanorod arrays. *Sci. Rep.* **6**, 30543; doi: 10.1038/srep30543 (2016).

## Supplementary Material

Supplementary Information
