# Supplementary material for: Enhanced photoelectrochemical and photocatalytic behaviors of MFe2O4 (M = Ni, Co, Zn and Sr) modified TiO2 nanorod arrays
Source: Sci Rep. 2016 Jul 28;6:30543. doi: 10.1038/srep30543 (PMC4964351; doi:10.1038/srep30543)
Supplement: Supplementary Information [file srep30543-s1.doc]

**supplementary information**

Enhanced photoelectrochemical and photocatalytic behaviors of MFe_2_O_4_ (M = Ni, Co, Zn and Sr) modified TiO_2_ nanorod arrays

**Xin Gao, Xiangxuan Liu, Zuoming Zhu, Xuanjun Wang, Zheng Xie***

*High-Tech Institute of Xi’an, Xi’an, 710025, China*

*Correspondence should be addressed to Zheng Xie; [xiezheng10@tsinghua.org.cn](mailto:xiezheng10@tsinghua.org.cn)

^＋^these authors contributed equally to this work


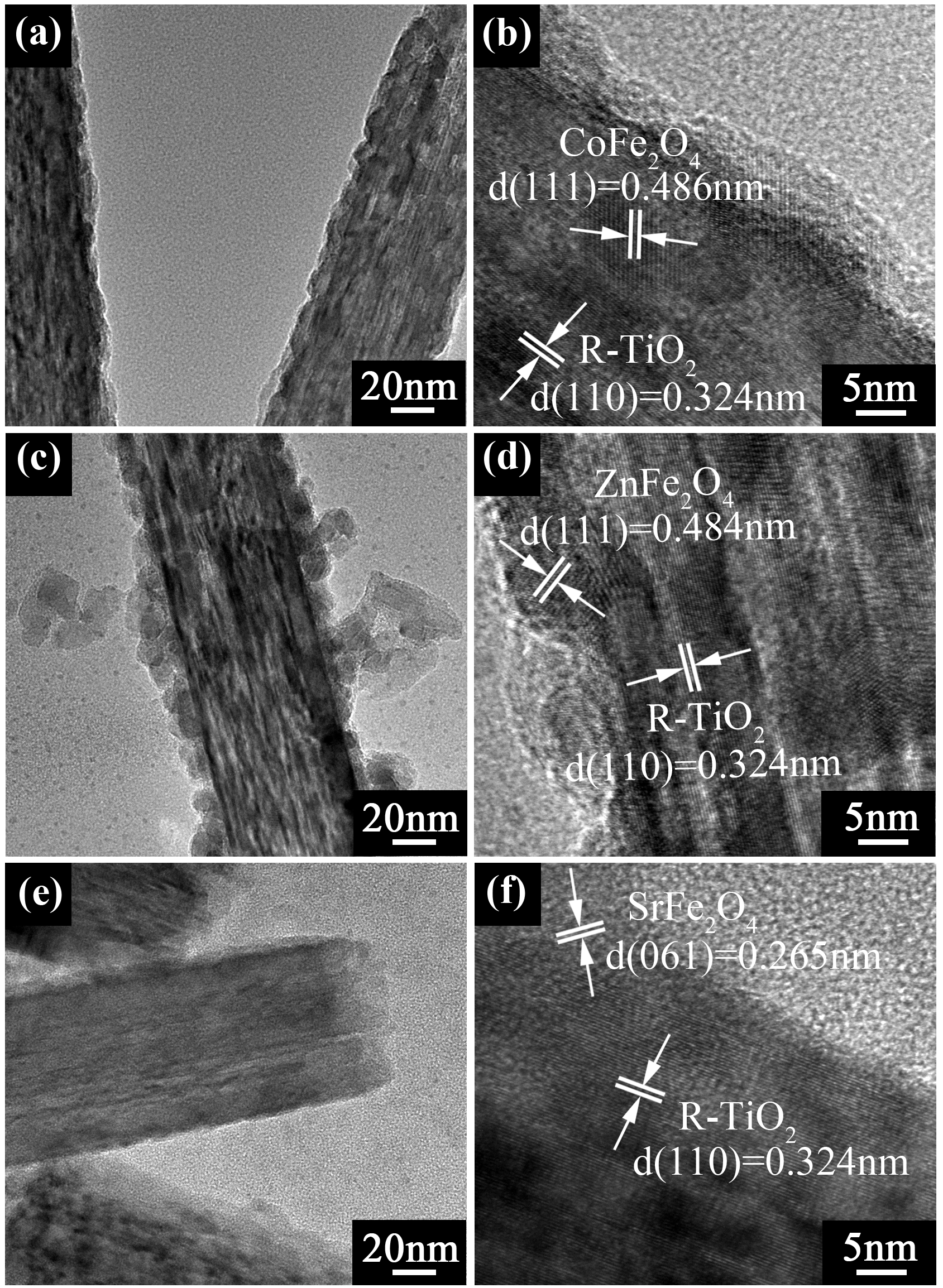


Supplementary Figure S1. TEM images of (a) CoFe_2_O_4_/TiO_2_ NRAs, (b) ZnFe_2_O_4_/TiO_2_ NRAs and (c) SrFe_2_O_4_/TiO_2_ NRAs
